# Celecoxib targets breast cancer stem cells by inhibiting the synthesis of prostaglandin E_2_ and down-regulating the Wnt pathway activity

**DOI:** 10.18632/oncotarget.23250

**Published:** 2017-12-14

**Authors:** Chaolin Huang, Yuanhong Chen, Hang Liu, Jing Yang, Xuejing Song, Junping Zhao, Na He, Chengji J. Zhou, Yongping Wang, Changjiang Huang, Qiaoxiang Dong

**Affiliations:** ^1^ Institute of Environmental Safety and Human Health, School of Laboratory Medicine and Life Science, Wenzhou Medical University, Wenzhou 325035, P.R. China; ^2^ Department of Biochemistry and Molecular Medicine, University of California at Davis, School of Medicine, Sacramento, CA 95817, USA

**Keywords:** CSCs, celecoxib, PGE_2_, Wnt pathway, EMT

## Abstract

Pharmacological targeting of breast cancer stem cells (CSCs) is highly promising for the treatment of breast cancer, as the small population of CSCs is responsible for tumor initiation, progression, recurrence and chemo-resistance. Celecoxib is one of the most commonly used non-steroidal anti-inflammatory drugs (NSAIDs), which have chemo-preventive activity against cancers, including breast cancer and colorectal cancer. However, the mechanisms by which NSAIDs exert its cancer prevention effects have yet been completely understood. In the present study, we investigated for the first time the effect of celecoxib on breast CSCs inhibition and its potential molecular mechanisms. Our results demonstrated that celecoxib suppresses CSC self-renewal, sensitizes chemo-resistance, inhibits epithelial to mesenchymal transition (EMT), and attenuates metastasis and tumorigenesis. Further exploring the underlying mechanism revealed that celecoxib targets breast CSCs by inhibiting the synthesis of prostaglandin E_2_ and down-regulating the Wnt pathway activity. Our findings suggest that celecoxib, by targeting CSCs, may be used as an adjuvant chemotherapy drug to improve breast cancer treatment outcomes.

## INTRODUCTION

Breast cancer is the second most common cancer in female patients, causing extensive mortality, psychological stress and health care burden worldwide [1–3]. Numerous strategies have been developed for the treatment of breast cancer over the past two decades, including chemotherapy, radiotherapy, hormonal therapy and immunological therapy [4]. However, disease progression, relapse and treatment resistance often resulted in treatment failures [5, 6], which raise the question of whether these conventional treatments have targeted the right tumor cells. Recently, it suggests that tumors contain a bulk of heterogeneous cells that derived from a small subset of cell population, which shows the characteristics of stem cell, termed cancer stem cell (CSC) or tumor-initiating cell (TIC) [7, 8]. CSCs contribute to tumor initiation, progression, metastasis, recurrence and treatment resistance [9]. Since the first identification of CSCs in leukemia, CSCs have been identified in almost all cancer types, including breast cancer, pancreatic cancer, lung cancer, gastric cancer and colorectal cancer [10–14]. Conventional cancer treatments are successful at killing the differentiated tumor cells but fail to eliminate CSCs [9]. Therefore, therapeutic strategies that target breast CSCs could potentially improve breast cancer treatment outcomes.

The Wnt pathway is an ancient and evolutionary conserved self-renewal pathway, which regulates stem cells to determine cellular fate during embryonic development and keep tissue homeostasis in adults [15]. The canonical Wnt pathway is mediated by β-catenin, a key intracellular mediator of the pathway, whose degradation is controlled by a proteasomal complex consisting of adenomatous polyposis coli (APC), glycogen synthase kinase 3β (GSK-3β) and AXIN. In the absence of Wnt ligands, cytosolic β-catenin levels are kept low by proteasomal degradation. In the presence of Wnt ligands, ligand-receptor binding induces the stabilization of β-catenin and promotes β-catenin nuclear accumulation. In the nucleus, β-catenin interacts with transcription factors of the LEF/TCF family and induces the expression of Wnt target genes such as *SURVIVIN*, *CYCLIN-D1*, *MMP-2*, *C-MYC* and *AXIN-2* [16]. Deregulation of Wnt pathway has been associated with various human cancers such as breast cancer, gastric cancer, and colorectal cancer [17]. Most importantly, previous studies have shown that CSCs require high Wnt signaling activity to maintain their self-renewal and tumorigenic properties, indicating that Wnt signaling pathway is a potential target for breast CSCs [18].

Celecoxib is one of the most commonly used non-steroidal anti-inflammatory drugs (NSAIDs) for the treatment of fever, pain, stiffness, and swelling. Numerous experimental and epidemiological studies have demonstrated that NSAIDs have chemo-preventive activity against cancers, including breast cancer and colorectal cancer [19–22]. Many case-control studies have also shown a significant decrease in the risk of breast cancer among women with regular NSAIDs use [23, 24]. However, the mechanisms by which NSAIDs exert its cancer prevention effects have yet been completely understood. The anti-inflammatory action of NSAIDs is mediated via their inhibitory effect on cyclooxygenase-2 (COX-2) activity and the synthesis of prostaglandin E_2_ (PGE_2_), while both COX-2 and PGE_2_ are strong inducers for inflammation. Earlier studies have shown that PGE_2_ was able to enhance the expansion of stem cells in the hematopoietic system and CSCs in colorectal tumors through the activation of Wnt pathway [25–30]. In the present study, we showed for the first time that celecoxib targets breast CSCs by inhibiting the synthesis of PGE_2_ and down-regulating the Wnt pathway activity.

## RESULTS

### Celecoxib suppresses breast cancer cell proliferation, CSC growth and self-renewal

**Figure 1:**
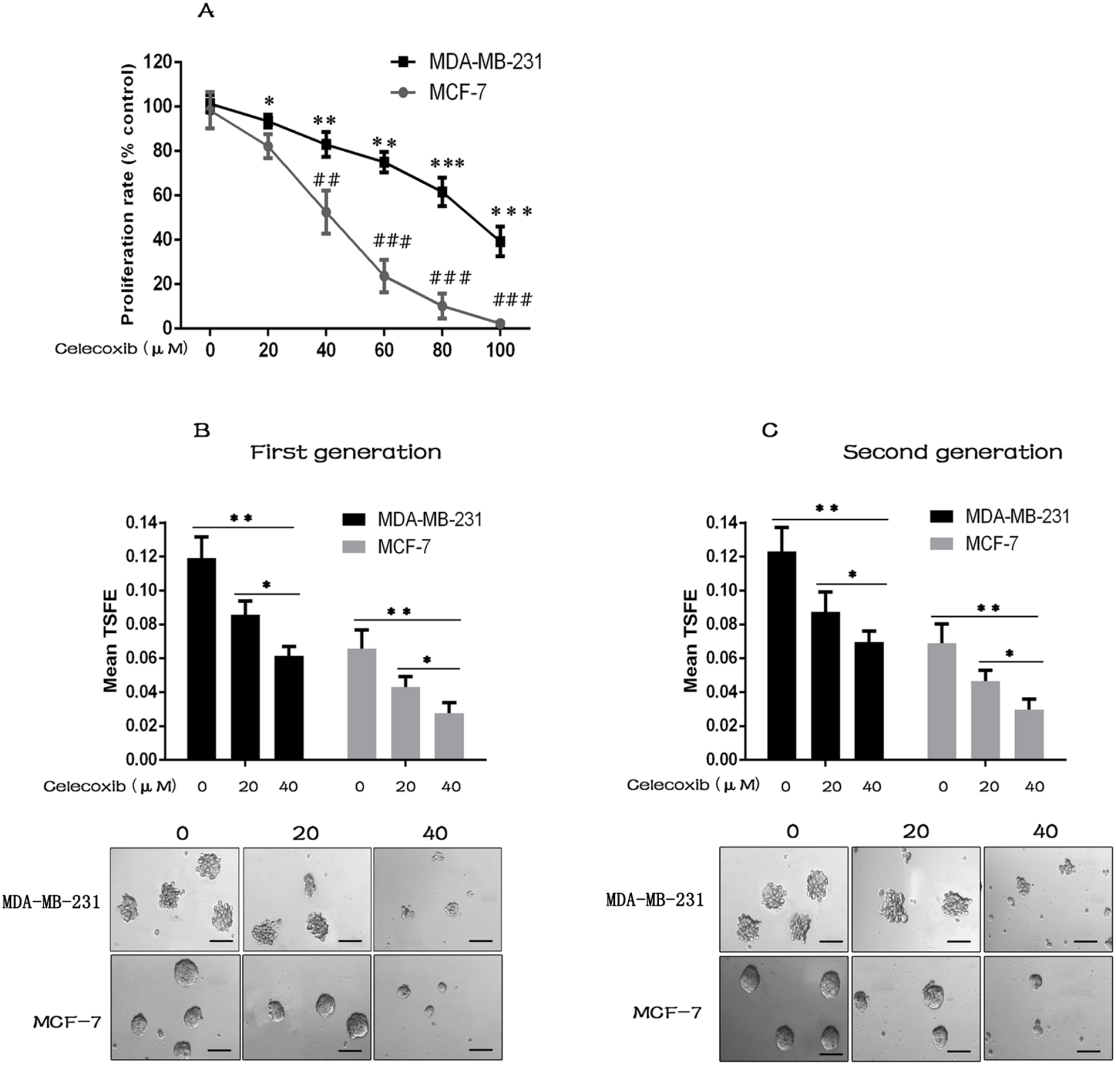
Celecoxib suppresses breast cancer cell proliferation, CSC growth and self-renewal **(A)** The cell proliferation of both MCF-7 and MDA-MB-231 cells was decreased by celecoxib treatment in a concentration dependent manner. **(B** and **C)** Celecoxib inhibited the first (with celecoxib treatment) and second (without additional celecoxib treatment) generation tumorsphere formation of both MAD-MB-231 and MCF-7 cells. The tumorsphere sizes greater than 100 μm were enumerated, and a representative image of tumorspheres is shown. TSFE: tumorsphere formation efficiency. Scale bar = 100 μm. Data are presented as the means ± SD from three independent experiments; ^*^, *P* < 0.05; ^**^, ^##^ *P* < 0.01; ^***^, ^###^ *P* < 0.001.

The cytotoxic effects of celecoxib on monolayer culture of breast cancer cell lines MCF-7 and MDA-MB-231 were evaluated by CCK-8 detection kit. MCF-7 cells are estrogen-positive and poorly invasiveness, while MDA-MB-231 cells are triple negative and highly invasiveness. Celecoxib inhibited cell proliferation of both MCF-7 and MDA-MB-231 cells in a concentration dependent manner (Figure 1A). Celecoxib inhibited MCF-7 cells more efficiently than that of MDA-MB-231 cells as the IC50 value was much lower in the former than that in the latter (Table 1).

**Table 1:** IC50 of different drugs / combinations (μM)

Drugs	MDA-MB-231	MCF-7
Celecoxib	89.05	40.05
Cisplatin	7.95	40.63
Cisplatin + 20 μM Celecoxib	4.18	21.40
5-FU	444.40	78.32
5-FU + 20 μM Celecoxib	112.00	20.16

Note: IC50 was calculated by nonlinear regression analysis using GraphPad Prism software.

The non-adherent tumorsphere formation assay is commonly used as an *in vitro* surrogate to quantify the frequency of CSCs [31, 32], and the ability of tumorspheres to be serially passaged at clonal density is an indirect marker of CSC self-renewal [33]. Treatment with celecoxib resulted in a significant reduction of tumorsphere formation efficiency (TSFE) of both MCF-7 and MDA-MB-231 cells in a dose dependent manner (Figure 1B). To evaluate the effect of celecoxib on CSC self-renewal, the primary tumorspheres were collected and dissociated into single cells. The single cells derived from treated or untreated primary tumorspheres were replated without continuous celecoxib exposure. The second generation TSFE was significantly lower in cells derived from celecoxib-treated primary tumorspheres as compared to cells derived from untreated primary tumorspheres (Figure 1C).

### Celecoxib sensitizes breast cancer cells to chemotherapeutic drugs by selectively targeting CSCs

**Figure 2:**
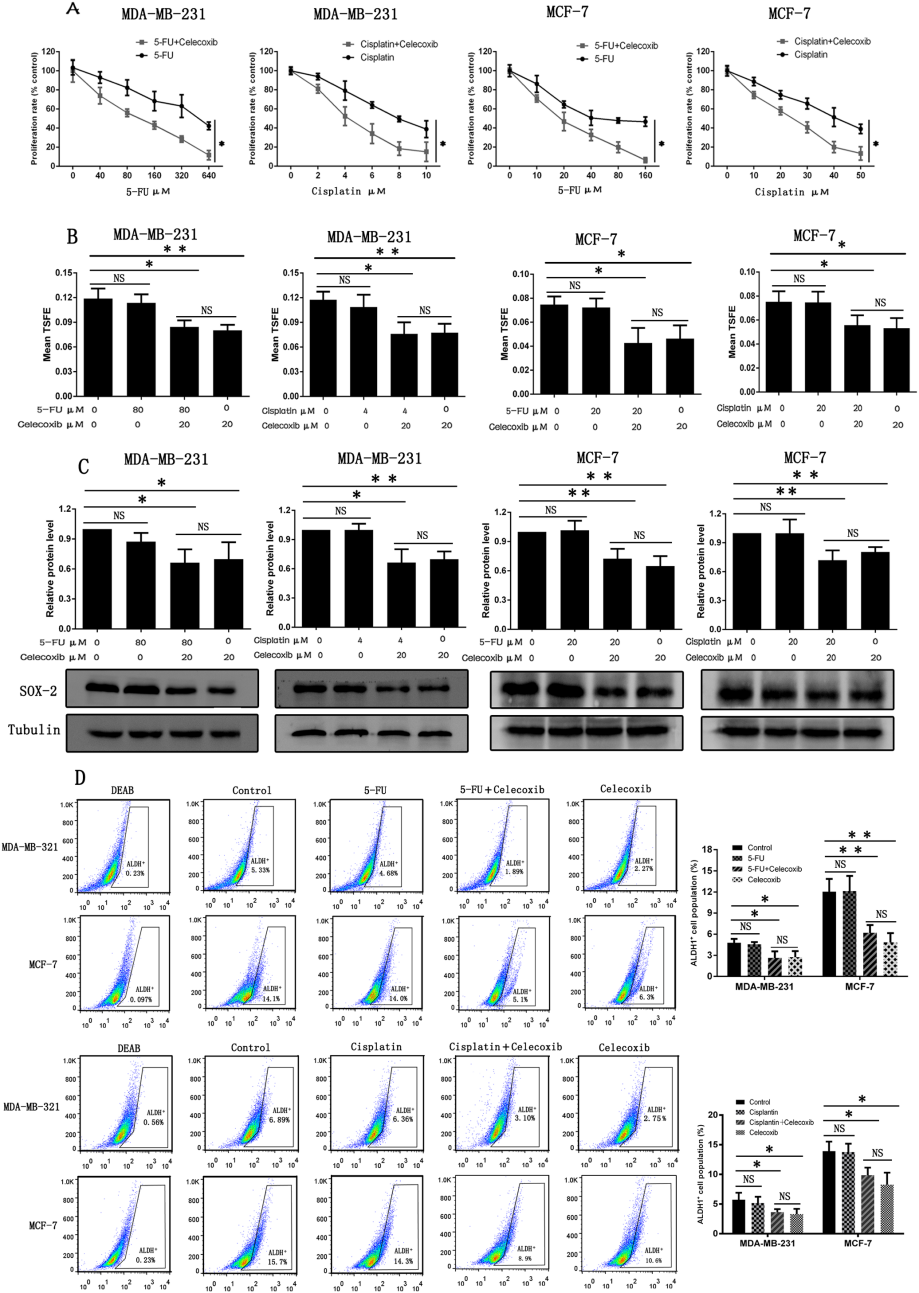
Celecoxib sensitizes breast cancer cells to chemotherapeutic drugs by selectively targeting CSCs **(A)** CCK-8 was performed to evaluate cell proliferation of both MDA-MB-231 and MCF-7 cells after being treated with various concentrations of chemotherapeutic drugs (5-FU, cisplatin) alone or in combination with 20 μM celecoxib. **(B**, **C** and **D)** Non-adherent tumorsphere formation assay, western-blot assay and flow cytometry analysis were performed to evaluate the effects of celecoxib on CSCs. MDA-MB-231: 5-FU, 80 μM; cisplatin, 4 μM; Celecoxib, 20 μM. MCF-7: 5-FU, 20 μM; cisplatin, 20 μM; Celecoxib, 20 μM. Data are presented as the means ± SD from three independent experiments; ^*^, *P* < 0.05; ^**^, *P* < 0.01; NS, Non statistical significance.

CSCs share many features of normal stem cells, including the relative quiescence, resistance to chemotherapeutic drugs and resistance to apoptosis. Conventional chemotherapeutic drugs are successful at killing the differentiated cancer cells but fail to eliminate CSCs, and leading to chemo-resistance and tumor relapse [34]. Therefore, a combination use of drugs targeting both differentiated cancer cells and CSCs was proposed to improve cancer treatment efficacy [35]. In this study, evaluation of chemotherapeutic drugs of 5-FU or cisplatin in combination with 20 μM celecoxib showed that celecoxib sensitized MCF-7 and MDA-MB-231 cells to 5-FU or cisplatin treatment (Figure 2A; Table 1).

We further examined whether celecoxib sensitized both MCF-7 and MDA-MB-231 cells to chemotherapeutic drugs was achieved by selectively targeting CSCs. Treatment with 5-FU or cisplatin alone failed to reduce TSFE of both MCF-7 and MDA-MB-231 cells, but co-treatment with celecoxib or treatment with celecoxib alone decreased the TSFE significantly, while no statistical difference was found between co-treatment and celecoxib treatment alone groups (Figure 2B). Previous studies have reported that SOX-2 is a CSC marker, which is used to identify and characterize CSCs in a variety of tumors including breast tumor [36–38]. Western-blot analysis revealed that 5-FU or cisplatin alone was unable to reduce SOX-2 expressions, but co-treatment with celecoxib or treatment with celecoxib alone led to a significant reduction of SOX-2 expression, while no statistical difference was found between co-treatment and celecoxib treatment alone groups (Figure 2C). In addition, we also identified CSCs based on their expression of high aldehyde dehydrogenase (ALDH) activity [39]. Flow cytometry analysis showed that 5-FU or cisplatin alone was unable to reduce the ALDH-positive cell population, but co-treatment with celecoxib or treatment with celecoxib alone led to a significant reduction of ALDH-positive cell population, while no statistical difference was found between co-treatment and celecoxib treatment alone groups (Figure 2D). Together, these findings indicate that celecoxib sensitizes breast cancer cells to chemotherapeutic drugs by selectively targeting CSCs.

### Celecoxib inhibits EMT gene signature

**Figure 3:**
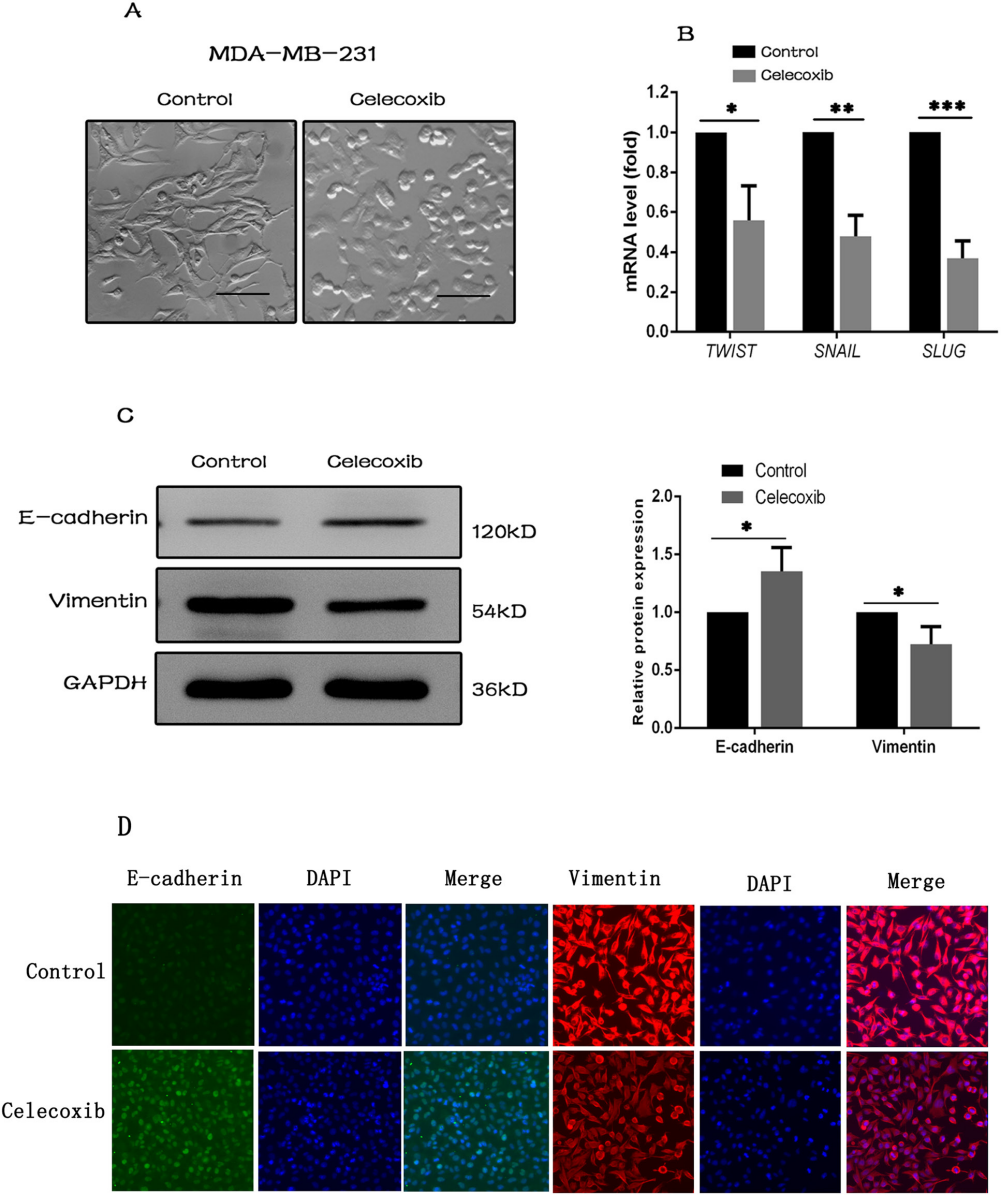
Celecoxib inhibits EMT gene signature **(A)** Morphological changes of MDA-MB-231 cells treated with celecoxib (magnification, ×100). **(B)** Relative mRNA expressions of mesenchymal makers including *SNAIL*, *SLUG*, and *TWIST* following celecoxib treatment. **(C** and **D)** The protein expression changes of E-cadherin and Vimentin following celecoxib treatment. Scale bar = 50 μm. Data are presented as the means ± SD from three independent experiments; ^*^, *P* < 0.05; ^**^, *P* < 0.01; ^***^, *P* < 0.001.

The epithelial to mesenchymal transition (EMT) is a basic process in morphogenesis of various tissues during embryonic development. Recent studies have suggested that EMT is also associated with generation of CSCs [40–43]. MDA-MB-231 cells typically have a spindle-shaped mesenchymal morphology, however, changed into cobble-stone-like epithelial appearance following celecoxib treatment (Figure 3A). To test the hypothesis that celecoxib may inhibit EMT, a group of mesenchymal makers including *SNAIL*, *SLUG* and *TWIST* was analysed by RT-PCR [44]. Our results showed that mRNA expression of these mesenchymal makers was decreased upon celecoxib treatment (Figure 3B). In addition, the expression of two EMT markers of E-cadherin and Vimentin were evaluated by western blot and immunofluorescence staining analysis. Both methods revealed that celecoxib induced an increase of E-cadherin along with a marked decrease of Vimentin in MDA-MB-231 cells (Figure 3C and 3D).

### Celecoxib attenuates breast cancer cell metastasis

**Figure 4:**
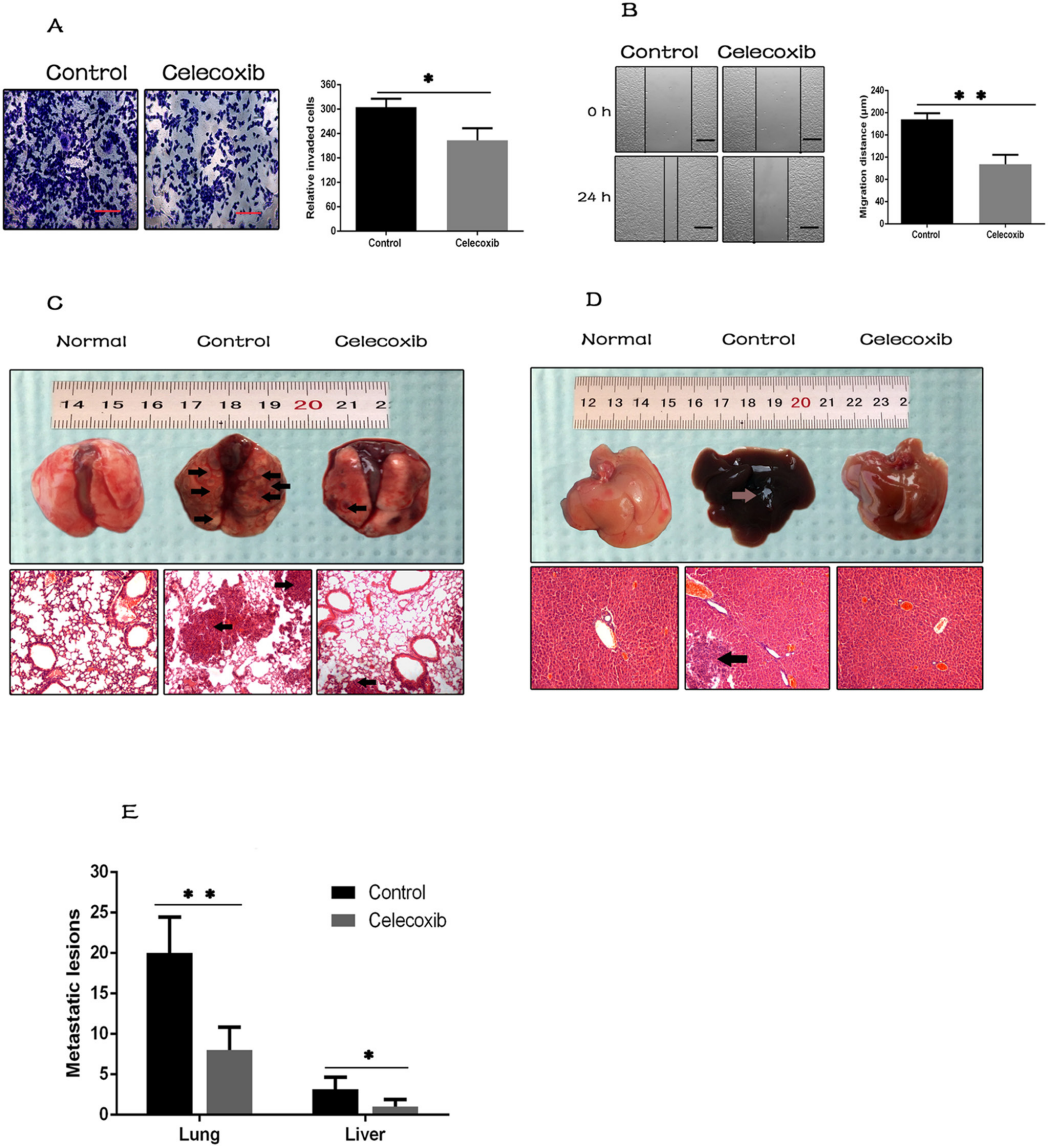
Celecoxib attenuates breast cancer cell metastasis **(A** and **B)** The effect of celecoxib on invasion and migration of MDA-MB-231 cells was measured by the transwell assay and wound healing assay respectively (magnification, ×100). **(C** and **D)** The effect of celecoxib on breast cancer cell metastasis was analyzed *in vivo*. Arrows indicate surface metastatic lesions on the lungs and livers, and metastatic lesions were confirmed by H&E staining (magnification, ×100). Normal lung and liver tissues were used as negative control. **(E)** Number of visible surface metastatic lesions on lungs and livers of individual mice were counted. Scale bar = 100 μm. Data are presented as mean ± SD. ^*^, *P* < 0.05; ^**^, *P* < 0.01; ^***^, *P* < 0.001.

It was reported that CSCs play a critical role in tumor metastasis [45, 46]. MDA-MB-231 cells were used to investigate the effect of celecoxib on breast cancer cell metastasis. Transwell migration assay and wound healing assay are commonly used to determine the metastatic property of cancer cells *in vitro*. In the transwell assay, celecoxib treatment led to a significant reduction of the number of cells invaded into the lower chambers (Figure 4A). Similarly, in the wound healing assay, celecoxib treatment attenuated the decreased distance between the wounded areas (Figure 4B).

We further evaluated the effects of celecoxib on breast cancer cell metastasis *in vivo* by tail vein injection mouse model. Twenty days post injection, the lungs and livers were harvested, and numbers of visible surface metastatic lesions were counted. Metastatic lesions were further confirmed by H&E staining (Figure 4C and 4D). It revealed that the number of surface metastatic lesions of both lungs and livers was significantly lower in celecoxib treated group than in the untreated control (Figure 4E).

### Celecoxib down-regulates the Wnt pathway activity by inhibiting the synthesis of PGE_2_ and reducing the phosphorylation of GSK-3β

**Figure 5:**
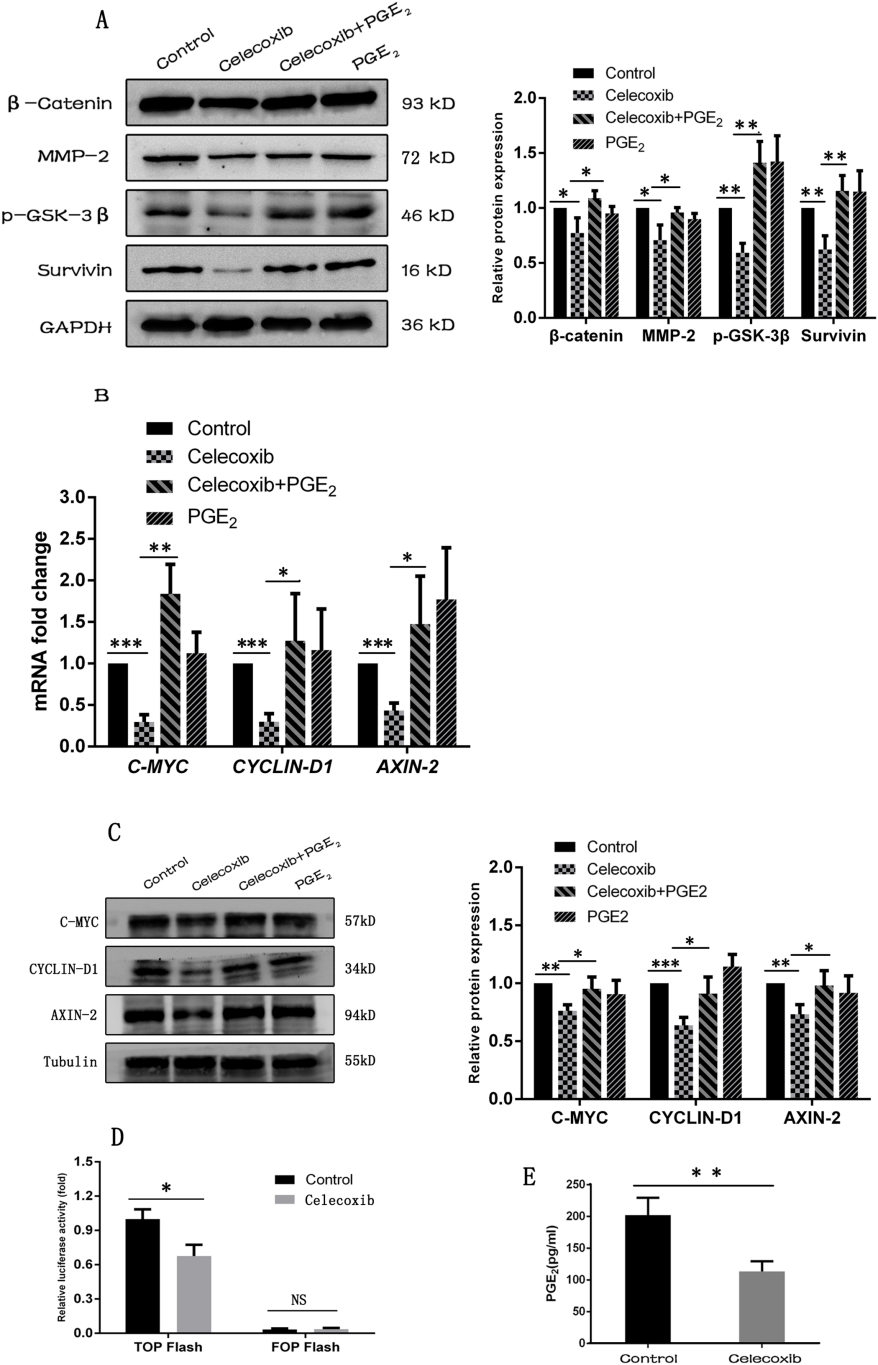
Celecoxib down-regulates the Wnt pathway activity by inhibiting the synthesis of PGE_2_ and reducing the phosphorylation of GSK-3β **(A**, **B** and **C)** The Wnt pathway target genes and components were measured by western-blot and RT-PCR. **(D)** Dual-luciferase reporter assay was carried out to evaluate the changes of Wnt pathway activity. **(E)** PGE_2_ levels in the cell culture media was measured by PGE_2_ ELISA kit. Celecoxib: 20 μM; PGE_2_: 20 nM. Data are presented as mean ± SD. ^*^, *P* < 0.05; ^**^, *P* < 0.01; ^***^, *P* < 0.001.

Wnt pathway has been implicated in the maintenance of CSCs in a variety of tumors [2, 17]. To explore whether celecoxib targets breast CSCs via down-regulating Wnt pathway activity, the Wnt pathway was investigated using MDA-MB-231 cells, which is known to express high Wnt pathway activity [47, 48]. Western blot analysis showed that celecoxib treatment decreased the expression of β-catenin, a Wnt pathway effective component, and Wnt pathway target proteins of Survivin and MMP-2 (Figure 5A). In addition, celecoxib decreased mRNA expression levels of Wnt pathway target genes including AXIN2 [49], CYCLIN-D1 [50] and C-MYC [51] (Figure 5B) and their corresponding protein expression levels (Figure 5C). We also use the dual-luciferase reporter assay to investigate the changes of Wnt pathway activity. Celecoxib decreased the luciferase activity of TOP flash reporter, which contains a TCF-binding site. In contrast, the FOP flash reporter containing mutated TCF-binding site, had very low luciferase activity and treatment with celecoxib didn't decrease the luciferase activity (Figure 5D). Together, these findings suggest that celecoxib down regulated Wnt pathway activity.

More importantly, celecoxib also decreased the synthesis of PGE_2_ in MDA-MB-231 cells (Figure 5E). To explore whether PGE_2_ was associated with celecoxib mediated Wnt pathway down-regulation, exogenous PGE_2_ was added to celecoxib treated MDA-MB-231 cells. As a result, the Wnt pathway activity was partially rescued by exogenous PGE_2_ addition, which was manifested by partially restored expression of β-catenin and Wnt pathway target genes [49–51] (Figure 5A–5C). Previous studies demonstrated that GSK-3β is a component of the destruction complex that renders β-catenin to proteasomal degradation and maintains Wnt pathway at a low activity. In addition, the destruction complex can be inactivated by the phosphorylation of GSK-3β at Ser9 [16]. In the present study, celecoxib reduced the phosphorylation of GSK-3β, while the addition of exogenous PGE_2_ increased the phosphorylation of GSK-3β (Figure 5A).

### Celecoxib inhibits tumorigenesis *in vivo* by inhibiting the synthesis of PGE_2_ and down-regulating the Wnt pathway activity

**Figure 6:**
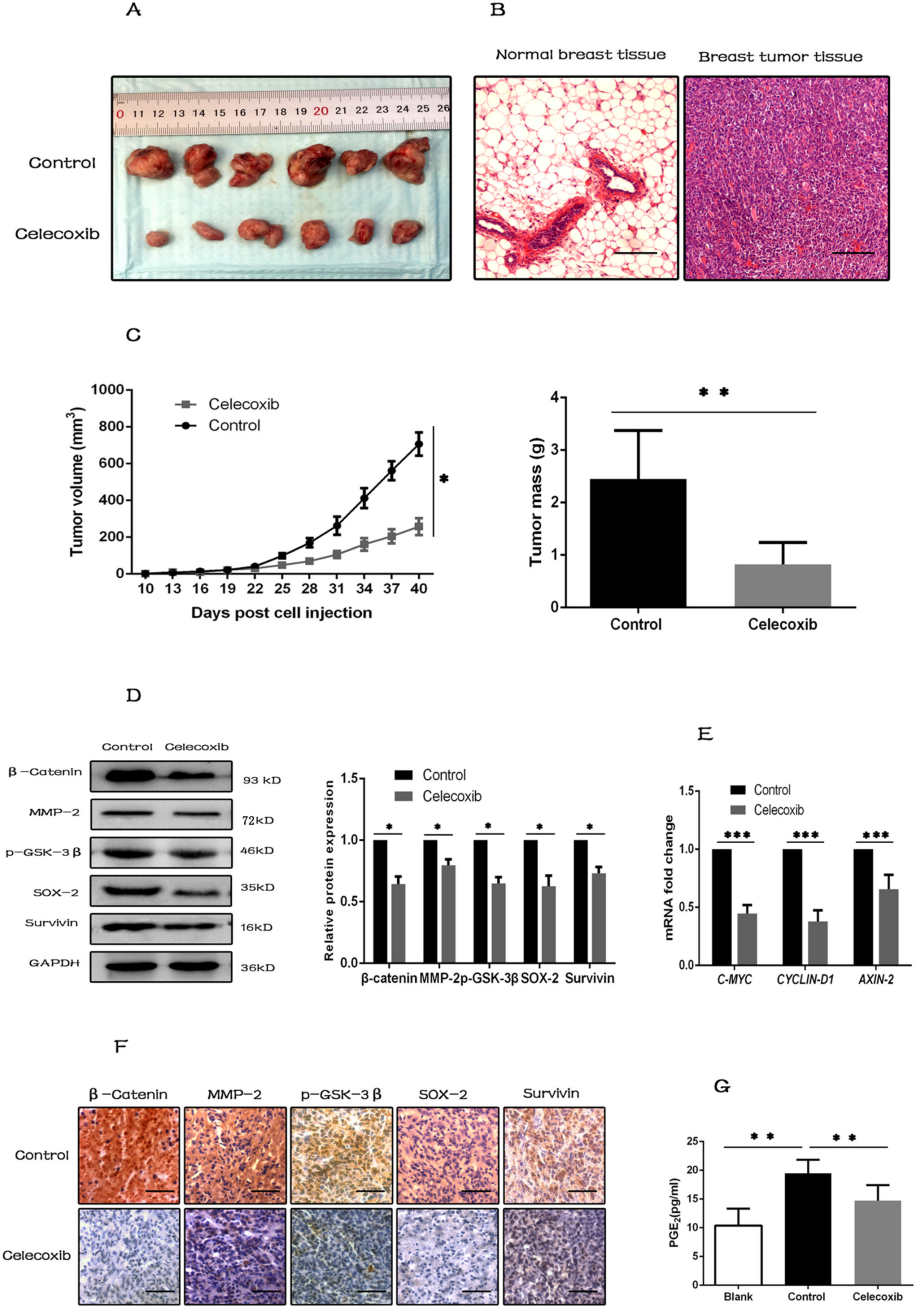
Celecoxib inhibits tumorigenesis *in vivo* by inhibiting the synthesis of PGE_2_ and down-regulating the Wnt pathway activity **(A)** Photographs of excised tumors from two groups of NOD/SCID mice (N = 6 per group). **(B)** Tumor tissues were confirmed by H&E staining, and normal breast tissues were used as negative control (magnification, ×100). **(C)** Tumor growth was measured with a caliper every three days, and tumors were weighted when the mice were sacrificed. **(D** and **E)** Proteins and RNAs were extracted from tumor tissues. Wnt pathway components (β-catenin, p-GSK-3β) and target genes (*MMP-2*, *Survivin*, *AXIN-2*, *CYCLIN-D1* and *C-MYC*), and CSC marker (SOX-2) were evaluated by western-blot or RT-PCR. **(F)** IHC staining for Wnt pathway components, target genes and CSC marker (SOX-2) in sections of tumor tissues (magnification ×200). **(G)** PGE_2_ levels in serum of the assayed animals were evaluated by PGE_2_ ELISA kit. Scale bar = 100 μm. Data are presented as mean ± SD. ^*^, *P* < 0.05; ^**^, *P* < 0.01; ^***^, *P* < 0.001.

Previous studies have shown that only CSCs, not the differentiated tumor cells, have the ability to initiate and sustain tumor growth [10, 52]. Xenograft mouse model was used to investigate the growth and tumorigenic property of breast CSCs *in vivo*. It showed that tumor growth was inhibited by celecoxib treatment, and tumor tissues were confirmed by H&E staining (Figure 6A–6C).

Celecoxib treatment also decreased the expression levels of Wnt pathway components and target genes, such as β-catenin, p-GSK-3β, MMP-2, Survivin, *AXIN2*, *CYCLIN-D1* and *C-MYC*, and the CSC marker SOX-2 in the xenograft tumor tissues (Figure 6D and 6E). These changes were also confirmed by immunohistochemistry of tumor tissues (Figure 6F).

Previous studies demonstrated that PGE_2_ was able to activate Wnt pathway by phosphorylating GSK-3β at Ser9 [28]. Therefore, PGE_2_ levels in serum of the assayed animals were evaluated by PGE_2_ ELISA kit. The results showed that PGE_2_ synthesis was low in serum of blank control mice, but was increased significantly in serum of tumor transplanted mice. However, this increase of PGE_2_ synthesis was inhibited by celecoxib treatment (Figure 6G).

## DISCUSSION

Accumulating evidences have suggested that tumors contain a bulk of heterogeneous tumor cells that derive from a small subset of cell population, which shows the characteristics of stem cells, termed as cancer stem cells (CSCs) [7, 8]. Besides, a critical role of CSCs in tumor initiation, progression, metastasis, and chemo-resistance has been well established in numerous cancer types including breast cancer [9]. Thus, the CSC concept has provided an important milestone in the understanding of chemo-resistance and cancer recurrence. On the basis of their characteristics, targeting and eradicating of CSCs using novel drugs represent a potential strategy for improving cancer treatment outcomes. In fact, previous studies have successfully identified some compounds such as salinomycin, curcumin, resveratrol, and niclosamide, which are able to target breast CSCs through the inhibition of Wnt pathway [53–57]. In addition, multiple lines of evidences demonstrate a significant reduction in breast cancer risk among women with regular NSAIDs use [23, 24]. However, its mechanism has yet been elucidated. In the present study, we demonstrate that celecoxib, a widely used NSAID, targets breast CSCs by inhibiting the synthesis of PGE_2_ and down-regulating the Wnt pathway activity.

Based on the ability that stem cells grow in serum-free non-adherent conditions, while differentiated cells fail to survive under such conditions. The non-adherent tumorsphere formation assay is commonly used as an *in vitro* surrogate assay to investigate the growth and tumorigenic property of breast CSCs [31, 32]. Using this assay, we found that celecoxib dramatically inhibited the tumorsphere formation of both MDA-MB-231 and MCF-7 cells (Figure 1B). In addition, the ability of tumorsphere to be serially passaged is an indirect marker of CSCs self-renewal [33]. Previous studies have shown that self-renewal is the key characteristic of breast CSCs, and the tumorsphere formation efficiency is retained as the serial passages [33]. In this study, we found that celecoxib treatment reduced MDA-MB-231 and MCF-7 cells subsequent secondary tumorsphere formation efficiency without additional treatment (Figure 1C). Furthermore, celecoxib completely depleted the tertiary tumorsphere formation (data not shown). These findings suggested that celecoxib is effective in inhibiting the growth, tumorigenesis and self-renewal of breast CSCs.

Previous studies have reported that conventional chemotherapeutic drugs are successful at killing the differentiated cancer cells but fail to eliminate CSCs, and leading to chemo-resistance and tumor relapse [34]. CSCs are resistant to conventional chemotherapeutic drugs via three possible mechanisms: 1) by increasing the expression of *ABC* transporter genes and consequently excluding chemotherapeutic drugs out of cells; 2) by activating DNA repair capacity and repairing the DNA damages inflicted by chemotherapies; and 3) by dividing infrequently and making them insensitive to antimitotic chemotherapeutic drugs [58, 59]. Based on these findings, we propose that combination use of drugs targeting both differentiated cancer cells and CSCs may improve cancer treatment outcomes [35]. In this study, we found that single use of conventional chemotherapeutic drugs (cisplatin and 5-FU) had a moderate effect on breast cancer cells but combination use of both celecoxib and conventional chemotherapeutic drugs dramatically increased the chemo-sensitivity of breast cancer cells (Figure 2A). This effect may be mediated via an additive mechanism that conventional chemotherapeutic drugs (5-FU and cisplatin) kill the differentiated cancer cells and celecoxib kills the CSCs. Next, we used the non-adherent tumorsphere formation assay and CSC marker SOX-2 to demonstrate that celecoxib decreased tumorsphere formation and SOX-2 expression, while the conventional chemotherapeutic drugs (cisplatin and 5-FU) were unable to do that (Figure 2B and 2C). In addition, previous studies have demonstrated that ALDH-positive cell population is enriched with CSCs [39]. In this study, we found that celecoxib decreased the ALDH-positive CSC population, while the conventional chemotherapeutic drugs (cisplatin and 5-FU) were unable to do that (Figure 2D). A recent study has also demonstrated that celecoxib abrogates chemo-resistance of bladder cancer cells by selectively targeting CSCs [60]. These findings indicate that celecoxib increases the chemo-sensitivity of breast cancer cells by selectively targeting CSCs.

The epithelial to mesenchymal transition (EMT) is a basic process in the morphogenesis of various tissues during embryonic development. It is defined that epithelial cells loss epithelial traits, acquire mesenchymal characteristics and show reduced intercellular adhesion and increased cell motility. Previous studies have suggested that EMT also associates with the generation of CSCs [40–43]. For example, the induction of EMT results in tumor aggressiveness, chemo-resistance and recurrence that are tightly linked with the characteristics of CSCs. In addition, various genes that induce EMT are also related to the expression of CSC markers. Furthermore, a high-throughput drug screening has identified salinomycin as a drug that specifically kills CSCs through the inhibition of EMT [53]. In this study, we observed that celecoxib induced morphological changes of MDA-MB-231 cells from spindle-shape to cobble-stone-like (Figure 3A). On the other hand, celecoxib decreased the mRNA expression of a panel of mesenchymal marker genes including *SNAIL*, *SLUG* and *TWIST* (Figure 3B). Moreover, western blot and immunofluorescence staining analysis demonstrated that celecoxib increased the level of epithelial marker protein of E-cadherin and decreased the level of mesenchymal marker protein of Vimentin (Figure 3C and 3D). These findings demonstrate that celecoxib is able to inhibit EMT, which is a property of breast CSCs.

Tumor metastasis is a complex process requiring the most aggressive tumor cells to survive the long time in the circulation system and form metastatic lesions in distance through invasion and migration. Previous studies have reported that CSCs display a mesenchymal morphology, show reduced intercellular adhesion and increased migration and invasion ability, therefore, promote tumor metastasis [45, 46]. Metastasis is a key feature of breast cancer, and extensive studies have demonstrated that breast CSCs play an important role in forming metastatic lesions. In the present study, we found that celecoxib dramatically inhibited both invasion and migration of MDA-MB-231 cells *in vitro* (Figure 4A and 4B). We also found that celecoxib reduced formation of metastatic lesions in lungs and livers *in vivo* (Figure 4C–4E). Given the critical role that CSCs play in tumor metastasis, it seems that celecoxib reduces breast cancer cell metastasis at least partially through the inhibition of breast CSCs.

It is well established that Wnt pathway regulates the self-renewal of stem cells in various organs, including mammary gland [15]. Ectopic activation of Wnt pathway in mouse models results in mammary carcinogenesis. In addition, Wnt pathway deregulation has been reported in breast cancer patients [17]. Most importantly, previous studies have shown that Wnt pathway plays a critical role in the maintenance of CSCs [18]. PGE_2_ is an inflammation inducer and is synthesized by COX-2. Previous studies have reported that PGE_2_ can enhance the expansion of stem cells in the hematopoietic system and CSCs in colorectal tumors through the up-regulation of Wnt pathway activity [25–30]. Another study also reported that paracrine and autocrine synthesized PGE_2_ induced the formation of CSCs through activation of Wnt pathway [61]. In this study, we demonstrate that celecoxib targets breast CSCs by inhibiting the synthesis of PGE_2_ and down-regulating the Wnt pathway activity.

In summary, we have used various approaches to comprehensively analyze the role of celecoxib plays in the inhibition of breast CSCs. Moreover, we have uncovered the molecular mechanism that celecoxib targets breast CSCs by inhibiting the synthesis of PGE_2_ and down-regulating the Wnt pathway activity. It conceptually advances the current understanding of the molecular mechanisms by which celecoxib acts on cancer prevention. Furthermore, gave the critical role that CSCs play in tumor initiation, progression, chemo-resistance and recurrence, it is interesting to speculate that celecoxib is useful not only in cancer prevention but also as an adjuvant drug to improve cancer treatment outcomes by targeting CSCs.

## MATERIALS AND METHODS

### Reagents and antibodies

Celecoxib, cisplatin, 5-fluorouracil (5-FU) and prostaglandin E_2_ (PGE_2_) were purchased from Sigma-Aldrich. Each compound was prepared as 1 mM stock solution in dimethylsulfoxide (DMSO) for dilution into various concentrations. The following mouse or rabbit monoclonal primary antibodies were used: anti-E-cadherin (Abcam), anti-MMP-2 (Abcam), anti-Vementin (Abcam), anti-c-MYC (Abcam), anti-Axin-2 (Abcam), anti-Cyclin-D1 (Abcam), anti- β-catenin (Cell Signaling Technology), anti-Survivin (Cell Signaling Technology), anti-SOX-2 (Cell Signaling Technology), anti-p-GSK-3β (Cell Signaling Technology), anti-GAPDH (Beyotime Biotechnology) and anti-Tubulin (Beyotime Biotechnology). HRP conjugated goat anti-mouse or anti-rabbit secondary antibodies were purchased from Beyotime Biotechnology.

### Monolayer cell culture

MDA-MB-231 and MCF-7 cells were purchased from American Type Culture Collection (ATCC) where they were characterized by mycoplasma detection, DNA Fingerprinting, isozyme detection and cell vitality detection. For monolayer Cell culture, human breast cancer cell lines MDA-MB-231 and MCF-7 were cultured in DMEM (Gibco) and RPMI-1640 (Gibco) respectively, which were supplemented with 10% fetal bovine serum (FBS) (Gibco), 100 U/ml penicillin and 100 U/ml streptomycin at 37°C in a humidified 5% CO_2_ incubator.

### Tumorsphere formation assay

For tumorsphere formation, single MDA-MB-231 and MCF-7 cells were suspended in serum-free DMEM-F12 medium, which was supplemented with B-27 (Invitrogen), 20 ng/ml EGF (Invitrogen), 20 ng/ml FGF (Invitrogen), 4 μg/ml heparin (Sigma-Aldrich) and plated at 2000 cells per well in a 96-well non-attachment plate (Thermo Fisher).

Primary tumorspheres were centrifuged (500 rcf), dissociated with trypsin, and then sieved through a 40 μm cell strainer to obtain single cell suspensions. These dissociated single cells were replated at 2000 cells per well in a 96-well non-attachment plate to form secondary tumorspheres.

Tumorspheres were cultured for seven days before counting the numbers. Individual tumorspheres were counted under an inverted microscope at ×100 magnification using the NIS-Elements imaging software. The percentage of cells capable of forming tumorspheres, termed as tumorsphere formation efficiency (TSFE), was calculated as follows: number of tumorspheres formed/number of single cells plated.

### Cell proliferation assay

The CCK-8 detection kit was used to assess cell proliferation according to the manufacturer’s instructions. Briefly, cells were seeded in a 96-well plate at 3000 cells per well. After incubation for 24 h, cells were treated with various concentrations of celecoxib for 24 h. Subsequently, CCK-8 solution was added and the plate was incubated at 37°C for 2.5 h. The number of viable cells was measured at a wavelength of 450 nm using a Versamax microplate reader.

For the evaluation of chemo-sensitivity, cells were treated with increasing concentrations of Cisplatin and 5-FU or in combination with 20 μM celecoxib. After 24 h of incubation, cell proliferation was assessed by CCK-8 detection kit as described above.

### Wound healing and transwell assay

Cell migration was determined by using a wound healing assay. Briefly, MDA-MB-231 cells were plated as monolayer at a density of 5×10^5^ cells per well in a 6-well plate and grown to confluence, then the monolayer cells were scratched with a 10-μL micropipette tip and then replaced with fresh medium supplemented with 20 μM celecoxib. After incubation for 24 h, the wound distances were measured using NIS-Elements imaging software.

Cell invasion was conducted by using Transwell Chambers (Corning). The upper chamber was coated with 100 μL Matrigel (Invitrogen), and the lower chamber was filled with 500 μL DMEM supplemented with 10% FBS. MDA-MB-231 cells were plated at 3000 cells per chamber in the upper chamber containing 20 μM celecoxib. The transwell chambers were incubated at 37°C and 5% CO_2_ for 24 h. Cells on the upper surface of the insert were removed using a cotton swab, and cells that had migrated to the lower surface were stained with 2% crystal violet (Beyotime Biotechnology) for 30 min. Images of migrated cells were taken and the number of migrated cells was counted under a microscope in three randomly selected fields (magnification 100x).

### Quantitative real-time PCR assay

To assess the expression levels of *SNAIL*, *SLUG*, *TWIST*, *CYCLIN-D1*, *C-MYC* and *AXIN-2*, total RNA was extracted from cultured cells or xenografted tumor tissues using the Trizol reagent (Life Technology) according to the manufacturer’s instructions. The quantity of RNA was determined by the spectrophotometer Nanodrop2000 (Thermal Fisher, USA). cDNA was prepared from 1 μg of total RNA using a Prim Script RT reagent kit (Takara, Japan) according to the manufacturer’s instructions. Primers for these genes were listed in Table 2. The relative changes in gene expression data were calculated by the 2^ΔΔCT^ method, β-actin was used as an internal control.

**Table 2:** The primers used for quantitative real-time PCR

Primers	Forward	Reverse
*SNAIL*	5′-GCCTAGCGAGTGGTTCTTCTGC-3′	5′-TGGTCGTAGGGCTGCTGGAA-3′
*SLUG*	5′-CCCTGGTTGCTTCAAGGACACA-3′	5′-GCTACACAGCAGCCAGATTCCT-3′
*TWIST*	5′-GGA GTCCGCAGTCTTACGAG-3′	5′-TCTGGAGGACCTGGTAGAGG-3′
*CYCLIN-D1*	5′-CGATGCCAACCTCCTCAACGA-3′	5′-TCCTCCTCGCACTTCTGTTCCT-3′
*C-MYC*	5′-CCCGCTTCTCTGAAAGGCTCTC-3′	5′-TCTGCTGCTGCTGCTGGTAGA-3′
*AXIN-2*	5′-GACCAAGCAGACGACGAAGCAT-3′	5′-CGTGCCTTTCCCATTGCGTTTG-3′
*β-ACTIN*	5′-CTGGAACGGTGAAGGTGACA-3′	5′-AAGGGACTTCCTGTAACAATGCA-3′

### Protein isolation and western blot assay

Cells or xenografted tumor tissues were lysed in lysis buffer (60 mM Tris-HCl, pH 6.8, 5% glycerol, 2% SDS and 1 mM PMSF). Protein concentrations were determined by BCA Protein Assay Kit (Beyotime Biotechnology). 30 μg of total proteins were separated by SDS-PAGE gels and transferred onto PVDF membranes. For immunoblotting, membranes were incubated with the following primary monoclonal antibodies: anti-β-catenin, anti-E-cadherin, anti-Vimentin, anti-MMP-2, anti-p-GSK-3β, anti-SOX-2, anti-c-MYC, anti-Axin-2, anti-Cyclin-D1 and anti-Survivin. Antibodies against Tubulin or GAPDH were used as internal controls. After washing three times in TBST, membranes were incubated with the corresponding HRP-conjugated secondary antibodies and visualized by the Gene Gnome imaging system. Relative protein expression quantities were analyzed by Quantity One software.

### PGE_2_ synthesis assay

The amounts of synthesized PGE_2_ in cell culture supernatants and serum of the assayed animals were measured by a commercial PGE_2_ detection kit (Cayman Chemicals) according to the manufacturer’s instructions.

### Immunohistochemistry assay and H&E staining

Formalin-fixed, and paraffin-embedded tumor, lung and liver tissues were sliced into 4 μm thick sections for immunohistochemistry assay or H&E staining. Endogenous peroxidase was inhibited by incubating sections with 3% H_2_O_2_ for 15 min and non-specific binding was blocked with 10% goat serum for 30 min at room temperature. Sections were incubated with specific primary antibodies at 4°C overnight, washed with PBST, and incubated with corresponding secondary antibodies for 1 h at room temperature. The sections were stained with diaminobenzidine (DAB) before observation under a microscope. The immunohistochemistry (IHC) intensity was scored by standard methods as described in previously published study [62].

### Immunofluorescence staining assay

MDA-MB-231 cells were grown in 24-well plates, and grown to 70% confluence prior to the treatment with 20 μM celecoxib or DMSO for 24 h. Cells were then fixed with 4% paraformaldehyde, permeabilized with 0.5% Triton X-100 and blocked with 10% goat serum for 1 h at room temperature. The cells were then incubated with the primary antibodies (mouse monoclonal antibody) anti-E-cadherin or anti-vimentin for overnight at 4°C. Alexa Fluor 488 or 647 conjugated goat anti-mouse were used as the secondary antibody. Finally the cells were washed 3 times in PBS and incubated with DAPI as the nuclear counterstain. Images were acquired at 100×magnification using a fluorescence microscope (Nikon, TE-2000U).

### Aldefluor assay

The Aldefluor assay was carried out according to the manufacturer’s instructions (Stem Cell Technologies, Canada). Briefly, MDA-MB-231 and MCF-7 cells were grown to 70% confluence prior to the treatment with 20 μM celecoxib or DMSO for 24 h. Single cells were suspended in Aldefluor assay buffer containing ALDH substrate and incubated for 45 minutes at 37°C. For negative control, half of the cell suspensions from each sample were incubated under the same conditions in the presence of DEAB (diethylaminobenzaldehyde). Flow cytometry was carried out to measure the ALDH-positive cell population. The data was analyzed by Flow Jo software.

### Dual-luciferase reporter assay

The MDA-MB-231 cells were grown to 80% confluence and transiently transfected with 1 μg of either TOP flash or FOP flash luciferase reporter vector and 20 ng of Renilla as internal control (Millipore Corporation) using 2 μL Lipo6000TM reagent (Beyotime Biotechnology) in each well of a 12-well plate. Five hours after transfection, the medium was replaced with completed cell culture medium containing 20 μM celecoxib or DMSO. Twenty-four hours later, cells were lysed in lysis buffer (Dual-luciferase Reporter Assay System, Promega, USA), and 20 μL of each sample was monitored for luciferase activity using a luminometer.

### Animal study

All animal experiments were performed according to protocols approved by the Committee of Care and Use of Laboratory Animals of Wenzhou Medical University, China. MDA-MB-231 cells (5 × 10^6^ per mouse) were transplanted into the cleared mammary fat pad of 8-week old NOD/SCID female mice (Shanghai Laboratory Animal Co., Shanghai, China). When tumors reached a palpable size, a total of 12 mice were randomly assigned to either control group or treatment group, six mice per group. Celecoxib dissolved in PBS (30 mg/kg/day) was administered by oral gavage every day for 30 days to the treatment group, and the same volume of PBS was given to the control group at the same time. In addition, three NOD/SCID female mice were used as blank control, which were neither transplanted with MDA-MB-231 cells nor administered with celecoxib. Tumor volumes were measured once every three days using a caliper, and calculated based on the following formula: volume = length×(width)^2^/2. All mice were euthanized at 30 days after the first drug treatment and the tumor masses were excised.

To investigate the effect of celecoxib on tumor metastasis, MDA-MB-231 cells (2 × 10^6^ per mouse) were injected into the tail vein of 12 NOD/SCID female mice. After injection, the mice were randomly assigned to the control or treatment group, six mice per group. Celecoxib was administered as described above. The mice were sacrificed at 20 days after first drug treatment and the lungs and livers were collected. Metastatic lesions on the surface of lungs and livers were confirmed by H&E staining and the number of lesions was counted.

### Statistical analysis

All data were presented as mean ± SD from three sets of independent experiments and analyzed using statistical software GraphPad Prism. Statistical differences between groups were determined by unpaired Student’s t-test or One-way Analysis of Variance (ANOVA); *P* < 0.05 was considered as statistical significance.

## Abbreviations

CSCs: cancer stem cells
EMT: epithelial to mesenchymal transition
5-FU: 5-fluorouracil
PGE_2_: prostaglandin E_2_
DMSO: dimethylsulfoxide
TSFE: tumorsphere formation efficiency.
